# Molecular detection and antimicrobial resistance of *Pseudomonas aeruginosa* from houseflies (*Musca domestica*) in Iran

**DOI:** 10.1186/s40409-015-0021-z

**Published:** 2015-05-30

**Authors:** Behsan Hemmatinezhad, Davood Ommi, Taghi Taktaz Hafshejani, Faham Khamesipour

**Affiliations:** Young Researchers and Elite Club, Shahrekord Branch, Islamic Azad University, Shahrekord, Iran; Functional Neurosurgery Research Center, Shahid Beheshti University of Medical Sciences, Tehran, Iran; Department of Clinical Sciences, Faculty of Veterinary Medicine, Shahrekord Branch, Islamic Azad University, Shahrekord, Iran

**Keywords:** Antimicrobial resistance, *Bla*TEM gene, Housefly (*Musca domestica*), Molecular detection, *Pseudomonas aeruginosa*

## Abstract

**Background:**

*Pseudomonas aeruginosa* is a common bacterium that can cause disease in humans and other animals. This study was conducted to screen for molecular detection and antimicrobial-resistant *P. aeruginosa* in *Musca domestica* in different locations in the Iranian provinces of Shahrekord and Isfahan.

**Methods:**

*Musca domestica* were captured by both manual and sticky trap methods, during the daytime, from household kitchens, cattle farms, animal hospitals, human hospitals, slaughterhouses and chicken farms at random locations in Shahrekord and Isfahan provinces of Iran, and subsequently transported to the laboratory for detection of *P. aeruginosa*. In the laboratory, flies were identified and killed by refrigeration in a cold chamber at −20 °C, then placed in 5 mL peptone water and left at room temperature for five hours before being processed. *Pseudomonas* isolates were preliminarily identified to genus level based on colony morphology and gram staining, and their identity was further confirmed by polymerase chain reaction.

**Results:**

Overall *bla*TEM gene was recovered from 8.8 % (53/600) of the *P. aeruginosa* isolated from houseflies collected from the two provinces. A slightly higher prevalence (10.7 %; 32/300) was recorded in Shahrekord province than Isfahan province (7.0 %; 21/300). The locations did not differ statistically (p < 0.05) in bacterial prevalence in flies. Seasonal prevalence showed a significantly lower infection frequency during autumn.

**Conclusions:**

Houseflies are important in the epidemiology of *P. aeruginosa* infections.

## Background

Houseflies (*Musca domestica*) are the most common of domestic flies. Their feeding and reproductive habits make them important mechanical and biological vectors of several human and veterinary pathogens including those causing nosocomial, enteric and anthropozoonotic infections [1–5]. They also serve as reservoirs and disseminators of metazoan parasites of both veterinary and human medical significance [2].

*Pseudomonas aeruginosa* is a fastidious multi-drug-resistant pathogen of veterinary and public health importance [6, 7]. The organism is involved in the etiology of some important emerging diseases; including nosocomial bloodstream infections and pneumonia [5–14]. The primary site of colonization and a frequent source of subsequent infection by *P. aeruginosa* is the gastrointestinal tract.

There are increasing reports of nosocomial infections associated with drug-resistant *P. aeruginosa* in Iran [14]. Meanwhile, there are many reports of *P. aeruginosa* disease outbreaks that are attributed to environmental sources [10, 15]. However, the role of such sources in sporadic *Pseudomonas* infections is not well defined [6]. There were also reported frequency of resistance and susceptible bacteria and fungi isolated from houseflies [5, 16]. A better understanding of the role of such environmental reservoirs in *Pseudomonas* infections would permit better use of strategies to minimize the transmission of the pathogen to vulnerable individuals. This study was conducted to screen for molecular detection and antimicrobial-resistant *P. aeruginosa* in *Musca domestica* in different locations in the Iranian provinces of Shahrekord and Isfahan.

## Methods

### Study area and sample collection

This study was conducted in Isfahan (32.6333° N, 51.6500° E) and Shahrekord (32.3256° N, 50.8644° E) provinces located in central and southwestern Iran, respectively. It involved collection of houseflies (n = 600) from household kitchens (n = 4), cattle farms (n = 4), chicken farms (n = 2), animal hospitals (n = 2), human hospitals (n = 4) and slaughterhouses (n = 2). The houseflies were captured by both manual and sticky trap methods. The fly samples were then transported to the laboratory of the Biotechnology Research Center, using separate sterile tubes to prevent cross-contamination between samples. In the laboratory, flies were identified and killed by refrigeration in a cold chamber at −20 °C. They were then placed in 5 mL peptone water and left at room temperature for five hours before being processed.

### Isolation of bacteria from fly samples

Bacteria were isolated from flies by placing them in a solution containing peptone water. Briefly, 500 μL of the peptone solution was inoculated in nutrient agar and blood agar plates. The inoculated plates were then incubated aerobically at 35 °C for 72 h. Bacterial colonies suggestive of *Pseudomonas* were sub-cultured and further incubated for seven days. Pure isolates were then maintained on the appropriate agar slant and stored at 28 °C.

### Identification of *Pseudomonas* and confirmation of the isolates

*Pseudomonas* isolates were preliminarily identified to genus level based on colony morphology and gram staining as previously reported [17, 18]. Following preliminary identification isolates were maintained in tryptic soy broth (TSB; Merck) for future use. Presumptive *Pseudomonas* isolates were confirmed by polymerase chain reaction using a method previously described [19].

### DNA isolation

DNA was extracted from the bacterial cells grown in TSB as described earlier by other authors [20, 21]. Briefly, bacterial cells were centrifuged at 15,000 × *g* for five minutes and washed in 1 mL of MNacl. The cells were then washed in 1 mL of TE buffer (50 mM Tris–HCl, pH 8.0, 50 nMEDTA), centrifuged again at high speed, and re-suspended in 0.7 mL of the same TE buffer. Two hundred micrograms of lysozyme (Sigma, USA) was then added, and the mixture incubated at 37 °C for one hour. The lysed cells were extracted twice with 1 mL of phenol-chloroform solution. DNA was precipitated from the aqueous phase with 0.33 MNH4- acetate and 2.5 volumes of cold ethanol overnight at −20 °C. The precipitated DNA was then dissolved in TE buffer. The quality of extracted DNA from samples was examined by electrophoretic analysis through a 1.5 % agarose gel.

### Polymerase chain reaction (PCR) Amplification of *bla*Tem-1gene

A pair of primers (Tem-F: 5′-TCCGCTCATGAGACAATAACC-3′ and Tem-R: 3′- ATAATACCGCACCACATAGCAG-5′) was designed to amplify the *bla*TEM gene in the extracted DNA using Genbank (Genbank, National Center for Biotechnology Information, www.ncbi.nlm.nih.gov/genbank). An amplification reaction yielding a PCR product of band size 296 bp was carried out in a total volume of 25 μL, consisting of 1 μM of each primer, 2 mM of MgCl_2_, 200 μM of dNTP, 5 μL of 10X PCR buffer, 1 U of Taq DNA polymerase (Fermentas, Germany) and 1 μg of template DNA. Distilled water was used as a negative control. Thermal PCR conditions consisted of five minutes of initial denaturation at 95 °C and then 30 cycles of denaturation at 94 °C for 60 s, annealing at 58 °C for one minute, and extension at 72 °C for one minute. Then followed a final extension at 72 °C for five minutes. The products were then maintained at 4 °C until processed. The amplified products were analyzed in 1.5 % agarose gel. Electrode buffer was TBE [Tris-base 10.8 g, 89 mM, boric acid 5.5 g, 2 mM, EDTA (pH 8.0) 4 mL of 0.5 M EDTA (pH8.0), with all components being combined in sufficient H_2_O and stirred to dissolve]. Gels were stained with ethidium bromide. Aliquots of 10 μL of PCR products were applied to the gel. Constant voltage of 80 for 20 min was employed to separate products. Subsequently, electrophoresis images were obtained in UVItec documentation systems (UK).

### Antimicrobial resistance testing

Antimicrobial resistance testing was performed by the Kirby-Bauer disc diffusion method on Mueller Hinton agar based on recommendations of CLSI (formerly the National Committee for Clinical Laboratory Standards – NCCLS) [22]. The following antibiotics were used in this study: ampicillin, amikacin, carbenicillin, cefalexin, ceftazidime, ceftriaxone, ceftizoxime, cefotaxime, ciprofloxacin, gentamicin, imipenem/cilastatin, norfloxacin, piperacillin, tobramycin and kanamycin (Pattan-Teb, Tehran, Iran).

### Statistical analysis

Data were analyzed by the statistical software SPSS® version 17.0 (SPSS Inc., USA). Frequencies of fly samples positive for *P. aeruginosa* and frequencies of isolates resistant to different antimicrobial agents were determined by computing descriptive statistics. Proportions were compared by chi-square test to determine statistical significance of the observed differences at p < 0.05.

## Results

**Fig. 1:**
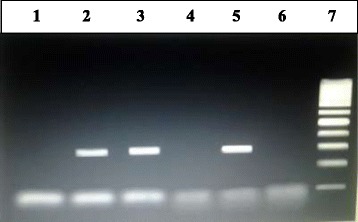
Agarose gel stained with ethidium bromide, for detection of TEM genes in *P. aeruginosa*. Lanes 1, 4 and 6 are negative. Lanes 2, 3 and 5 are positive tests for TEM (296 bp). Line 7 is a DNA ladder (Fermentas, Germany)

The overall prevalence of *P. aeruginosa* in houseflies in this study was 8.8 % (53/600) from houseflies collected from the two provinces. The expected size of amplicons for TEM genes in *P. aeruginosa* is 296 bp (Fig. 1). The 10.7 % (32/300) prevalence of flies obtained in Shahrekord province was slightly higher than the 7.0 % (21/300) found in Isfahan province. The locations and specific seasonal prevalence of *P. aeruginosa* are shown in Tables 1 and 2. There were no statistically significant differences (p < 0.05) in the bacterial prevalence in flies from the different locations. Furthermore, seasonal prevalence showed that a significantly lower frequency of infection occurred during autumn.

**Table 1:** Recovery frequencies of Pseudomonas aeruginosa from houseflies captured at different locations in Shahrekord and Isfahan provinces of Iran

Location	Proportion of *Pseudomonas aeruginosa* in % (n)
Kitchens (n = 4)	4.0 (100)
Cattle farms (n = 4)	15.0 (100)
Chicken farms (n = 2)	5.0 (100)
Slaughterhouses (n = 2)	6.0 (100)
Animal hospitals (n = 2)	14.0 (100)
Human hospitals (n = 4)	9.0 (100)

**Table 2:** Recovery frequencies of Pseudomonas aeruginosa from houseflies captured during different seasons in Shahrekord and Isfahan provinces of Iran

Season	Proportion of *Pseudomonas aeruginosa* in % (n)
Spring	10.7 (150)
Summer	14.7 (150)
Autumn	3.3 (150)
Winter	10.0 (150)

Antimicrobial resistance profiles of *P. aeruginosa* isolates obtained in this study are displayed in Table 3.

**Table 3:** Antimicrobial resistance profiles of P. aeruginosa isolates against 15 antimicrobial agents

Antimicrobial agent	Proportion of resistant isolates (%)
Ampicillin	100.0
Amikacin	64.2
Carbenicillin	69.8
Cefalexin	100.0
Ceftazidime	71.7
Ceftriaxone	100.0
Ceftizoxime	71.7
Cefotaxime	100.0
Ciprofloxacin	58.5
Gentamicin	49.1
Imipenem/cilastatin	49.1
Norfloxacin	45.3
Piperacillin	60.4
Tobramycin	56.6
Kanamycin	100.0

## Discussion

The role of houseflies as reservoirs of infectious microorganisms has been described by several researchers [23–25]. Because of their habitat preferences, mobility, feeding habits, and attraction to residential areas, these flies have a great potential to disseminate bacterial pathogens, including those responsible for causing human and animal infections [26, 27]. Flying back and forth between different sites, the flies transmit the pathogens to surrounding communities both mechanically, via contaminated mouthparts and legs; and biologically, via excretion of ingested microbes [28, 29]. In the present study, 8.8 % (53/600) of the houseflies collected at the different locations were positive for *Pseudomonas* spp. The location-specific prevalence of the pathogen among the collected flies ranged from 4.0 % for those collected in kitchens to 15.0 % for flies captured on cattle farms. Both the overall and site-specific prevalence of *Pseudomonas* spp. among housefly population sampled in the current study were lower than the values reported elsewhere [30]. This observation highlights differences in contamination rates with the pathogen in the sampling locations in these different studies.

A significant proportion (9.0 %) of houseflies captured in the hospital environment carried *Pseudomonas* spp. This finding corroborates the suggestion by Fotedar *et al.* [1] that there is a high chance that flies in hospital settings would become contaminated with pathogenic microorganisms as the microorganisms is widespread in such an environment. The implication of this is that, in turn, the flies contaminate the patient environment so that patients are exposed to healthcare associated infections [1]. Different studies have demonstrated *P. aeruginosa* nosocomial outbreaks caused by environmental sources [6, 31, 32].

This study recorded high prevalence of *P. aeruginosa* in samples collected from cattle farms and animal hospital environments. The observation is similar to other reported incidences of bacterial fly infestations in a number of studies [8, 26, 29, 33, 34]. This finding may be attributable to the presence of organic waste in and around these facilities, which provide excellent habitats for the growth and development of both bacterial pathogens and these insect pests [35]. Researchers point out that all environments rich in decomposing organic matter harbor diverse microbes and serve as suitable substrate for development of houseflies and other filth flies [5, 14].

The samples collected from the kitchen environment showed the lowest prevalence (4.0 %) of *P. aeruginosa* in this study. A similarly low prevalence (3.3 %) of this pathogen among houseflies sampled in kitchens was found by Jia *et al*. [36]. The findings in these two different studies would suggest a low level of contamination of the kitchen environment by this bacterium, a diminution that may be linked to hygienic practices in kitchens in attempts to produce safe food.

This finding that houseflies carry antimicrobial-resistant bacteria including *P. aeruginosa* has been previously reported in Iran and elsewhere [16, 37–39]. Generally, *P. aeruginosa* isolates recovered from houseflies in the current study were highly resistant to all 15 antimicrobials tested, with the percentage of resistant isolates ranging from 45.3 % to 100.0 %. These included high resistance levels to imipenem (49.1 %) and amikacin (64.2 %) which were found by other authors to be effective against the bacterium [39]. The emergence of bacterial strains resistant to carbapenems, which are among the most effective antimicrobial agents against gram-positive and gram-negative bacteria, is suggested to be due to plasmid or integron-mediated carbapenemases, efflux systems, reduced porin expression and increased chromosomal cephalosporinase activity [40]. Unfortunately, there has been little consideration of arthropod vectors in the current control strategies for *P. aeruginosa* [41].

## Conclusions

We have detected the presence of *P. aeruginosa* from a significant proportion of *Musca domestica* sampled from random human and animal locations in Iran. Since a housefly can serve both as a mechanical and biological vector of *P. aeruginosa*, this finding indicates a risk to vulnerable humans and animals that is heightened by the occurrence of antimicrobial resistance among the isolates, which limits therapeutic options for the treatment of infections caused by the bacterium. Houseflies are important in the epidemiology of *P. aeruginosa* infections. Thus, future programs aimed at stemming infections caused by these organisms should take flies into account.

### Ethics committee approval

The present study was approved by the Ethics Committee of the Shahrekord Branch, Islamic Azad University.

## References

[1] Fotedar R, Banerjee U, Singh S, Shriniwas V, Verma AK (1992). The housefly (*Musca domestica*) as a carrier of pathogenic microorganisms in a hospital environment. J Hosp Infect..

[2] Förster M, Klimpel S, Sievert K (2009). The house fly (*Musca domestica*) as a potential vector of metazoan parasites caught in a pig-pen in Germany. Vet Parasitol.

[3] Blunt R, McOrist S, McKillen J, McNair I, Jiang T, Mellits K (2011). House fly vector for porcine circovirus 2b on commercial pig farms. Vet Microbiol.

[4] Nielsen AA, Skovgard H, Stockmarr A, Handberg KJ, Jorgensen PH (2011). Persistence of low-pathogenic avian influenza H5N7 and H7N1 subtypes in house flies (Diptera: Muscidae). J Med Entomol.

[5] Davari B, Khodavaisy S, Ala F (2012). Isolation of fungi from housefly (*Musca domestica* L.) at Slaughter House and Hospital in Sanandaj, Iran. J Prev Med Hyg.

[6] Kerr KG, Snelling AM (2009). *Pseudomonas aeruginosa*: a formidable and ever-present adversary. J Hosp Infect.

[7] Durojaiye OC, Carbarns N, Murray S, Majumdar S (2011). Outbreak of multidrug-resistant *Pseudomonas aeruginosa* in an intensive care unit. J Hosp Infect.

[8] Bradford PA (2001). Extended-spectrum ß-Lactamases in the 21st Century: characterization, epidemiology, and detection of this important resistance threat. Clin Microbiol Rev.

[9] Souli M, Galani I, Giamarellou H (2008). Emergence of extensively drug-resistant and pandrug-resistant Gram-negative bacilli in Europe. Euro Surveill.

[10] Floret N, Bertrand X, Thouverez M, Talon D (2009). Nosocomial infections caused by *Pseudomonas aeruginosa*: exogenous or endogenous origin of this bacterium?. Pathol Biol (Paris).

[11] Caselli D, Cesaro S, Ziino O, Zanazzo G, Manicone R, Livadiotti S (2010). Multidrug resistant *Pseudomonas aeruginosa* infection in children undergoing chemotherapy and hematopoietic stem cell transplantation. Haematologica.

[12] Yang MA, Lee J, Choi EH, Lee HJ (2011). *Pseudomonas aeruginosa* bacteremia in children over ten consecutive years: analysis of clinical characteristics, risk factors of multi-drug resistance and clinical outcomes. J Korean Med Sci.

[13] Essayagh T, Zohoun A, Essayagh M, Elameri A, Zouhdi M, Ihrai H (2011). Bacterial epidemiology in the burns unit at military teaching hospital Mohamed V of Rabat. Ann Biol Clin (Paris).

[14] Yaslianifard S, Mobarez AM, Fatolahzadeh B, Feizabadi MM (2012). Colonization of hospital water systems by *Legionella pneumophila*, *Pseudomonas aeroginosa*, and *Acinetobacter* in ICU wards of Tehran hospitals. Indian J Pathol Microbiol.

[15] Benitez L, Ricart M (2005). Pathogenesis and environmental factors in ventilator-associated pneumonia. Enferm Infecc Microbiol Clin.

[16] Davari B, Kalantar E, Zahirnia A, Moosa-Kazemi SH (2010). Frequency of resistance and susceptible bacteria isolated from houseflies. Iran J Arthropod Borne Dis.

[17] Pence MA, McElvania TeKippe E, Burnham CA (2013). Diagnostic assays for identification of microorganisms and antimicrobial resistance determinants directly from positive blood culture broth. Clin Lab Med.

[18] Weiser R, Donoghue D, Weightman A, Mahenthiralingam E (2014). Evaluation of five selective media for the detection of *Pseudomonas aeruginosa* using a strain panel from clinical, environmental and industrial sources. J Microbiol Methods.

[19] Begum S, Salam MA, Alam KF, Begum N, Hassan P, Haq JA (2013). Detection of extended spectrum β-lactamase in *Pseudomonas* spp. isolated from two tertiary care hospitals in Bangladesh. BMC Res Note.

[20] Clarke L, Millar BC, Moore JE (2013). Extraction of genomic DNA from *Pseudomonas aeruginosa*: a comparison of three methods. Br J Biomed Sci.

[21] Valadbeigi H, Tabatabaei RR, Malek A, Sekawi Z, Raftari M, Parvaneh K (2014). Genomic diversity and virulence genes among clinical isolates of *Pseudomonas aeruginosa*. Clin Lab.

[22] Clinical and Laboratory Standards Institute (2008). Performance standards for antimicrobial disk and dilution susceptibility tests for bacteria isolated from animals; approved standard. Second Informational Supplement.

[23] Rosef O, Kapperud G (1983). House flies (*Musca domestica*) as possible vectors of *Campylobacter fetus* subsp. *jejuni*. Appl Environ Microbiol.

[24] Holt PS, Geden CJ, Moore RW, Gast RK (2007). Isolation of *Salmonella enterica* serovar Enteritidis from houseflies *(Musca domestica)* found in rooms containing *Salmonella* serovar Enteritidis-challenged hens. Appl Environ Microbiol.

[25] Gupta AK, Nayduch D, Verma P, Shah B, Ghate HV, Patole MS (2012). Phylogenetic characterization of bacteria in the gut of house flies (*Musca domestica* L.). FEMS Microbiol Ecol.

[26] Graczyk TK, Knight R, Gilman RH, Cranfield MR (2001). The role of non-biting flies in the epidemiology of human infectious diseases. Microbes Infect.

[27] Zurek L, Gorham JR, Voeller JG (2008). Insects as vectors of foodborne pathogens. Wiley handbook of science and technology for Homeland security.

[28] Hui YH (2006). Handbook of food science, technology and engineering.

[29] Joyner C, Mills MK, Nayduch D (2013). *Pseudomonas aeruginosa* in *Musca domestica* L.: temporospatial examination of bacteria population dynamics and house fly antimicrobial responses. PLoS One.

[30] Rahuma N, Ghenghesh KS, Ben Aissa R, Elamaari A (2005). Carriage by the housefly (*Musca domestica*) of multiple-antibiotic-resistant bacteria that are potentially pathogenic to humans, in hospital and other urban environments in Misurata. Libya Ann Trop Med Parasitol.

[31] Diaz Granados CA, Jones MY, Kongphet-Tran T, White N, Shapiro M, Wang YF (2009). Outbreak of *Pseudomonas aeruginosa* infection associated with contamination of a flexible bronchoscope. Infect Control Hosp Epidemiol.

[32] Lanini S, D’Arezzo S, Puro V, Martini L, Imperi F, Piselli P (2011). Molecular epidemiology of a *Pseudomonas aeruginosa* hospital outbreak driven by a contaminated disinfectant-soap dispenser. PLoS One.

[33] Greenberg B (1971). Flies and diseases: ecology, classification and biotic association.

[34] Harwood RF, James MT (1979). Entomology in human and animal health.

[35] Ahmad A, Ghosh A, Schal C, Zurek L (2011). Insects in confined swine operations carry a large antibiotic resistant and potentially virulent enterococcal community. BMC Microbiol.

[36] Jia H, Liu X, Miao Y, Xia D, Pang T, Yu T, et al. Primary study on the outbreak of overwhelming housefly (*Musca domestica*) in kitchen. J Beijing Univ Agr. 2011; 1: CJFDTOTAL-BNXB201101004.

[37] Mugnier P, Dubrous P, Casin I, Arlet G, Collatz E (1996). A TEM-derived extended-spectrum beta-lactamase in *Pseudomonas aeruginosa*. Antimicrob Agents Chemother.

[38] Bertrand X, Thouverez M, Talon D, Boillot A, Capellier G, Floriot C (2001). Endemicity, molecular diversity and colonisation routes of *Pseudomonas aeruginosa* in intensive care units. Intensive Care Med.

[39] Said KB, Al-Jarbou AN, Alrouji M, Al-harbi HO (2014). Surveillance of antimicrobial resistance among clinical isolates recovered from a tertiary care hospital in Al Qassim. Saudi Arabia Int J Health Sci.

[40] Papp-Wallace KM, Endimiani A, Taracila MA, Bonomo RA (2011). Carbapenems: past, present, and future. Antimicrob Agents Chemother.

[41] Zimmer CR, de Castro LL D, Pires SM, Delgado Menezes AM, Ribeiro PB, Leivas Leite FP (2013). Efficacy of entomopathogenic bacteria for control of *Musca domestica*. J Invertebr Pathol.

